# Prevalence of Peripheral Artery Disease and Its Association With Coronary Artery Disease in Patients Undergoing Coronary Angiogram: A Single-Center Study

**DOI:** 10.7759/cureus.110642

**Published:** 2026-06-11

**Authors:** Mahilini Vipulan, Kumanan Thirunavukarasu, Guruparan Mahesan, Pethirupillai A Coonghe, Rajeshkannan Nadarajah

**Affiliations:** 1 Acute Medicine, Queen Elizabeth Hospital King's Lynn NHS Foundation Trust, King's Lynn, GBR; 2 Medicine, Faculty of Medicine, University of Jaffna, Jaffna, LKA; 3 Cardiology, Teaching Hospital Jaffna, Jaffna, LKA; 4 Community and Family Medicine, University of Jaffna, Jaffna, LKA; 5 Family Medicine, Wentworthville Medical and Dental Centre, Sydney, AUS

**Keywords:** ankle-brachial pressure index, atherosclerosis, coronary artery disease, peripheral artery disease, positive predictive value

## Abstract

Background

Peripheral artery disease (PAD) is an underrecognized manifestation of atherosclerotic cardiovascular disease compared with coronary artery disease (CAD) and cerebrovascular diseases despite sharing a common atherosclerotic pathogenesis and many of the risk factors. Although a coronary angiogram remains the gold standard for diagnosing CAD, its invasive nature limits its suitability as a screening tool. The ankle-brachial pressure index (ABPI) has been recognized as a reliable marker of peripheral arterial disease and generalized atherosclerosis; however, its utility in predicting CAD in patients undergoing a coronary angiogram requires further evaluation.

Objective

This study aimed to determine the prevalence of PAD by using the ABPI and to study the association of CAD in patients undergoing a coronary angiogram.

Methodology

This cross-sectional analytical study was conducted among 431 patients who underwent a coronary angiogram at the cardiology unit of the Teaching Hospital in Northern Sri Lanka from October 2022 to May 2023. Patients who consented to participate in the study were recruited consecutively until reached sample size. All collected data were entered into the Statistical Package for the Social Sciences (SPSS) software, version 30.0 (IBM Corp., Armonk, NY), and analyzed. Measurement of ABPI was performed to confirm the diagnosis of PAD.

Results

The majority of patients (74%) were male, and the mean age was 59.34 ± 10.19 years, with a range of 31 to 81 years. Prevalence of PAD in patients undergoing a coronary angiogram was 12.5% (95% CI: 9.6-15.9). Among 54 patients with PAD, 51 (94.4%) had CAD. This association is statistically significant (p=0.019). Patients with PAD have a 3.8 times higher risk (95% CI: 1.3-15.8) of having CAD. Specificity of PAD for predicting CAD (ABPI<0.9) was 95.8% (95% CI: 88.4%-98.6%), and sensitivity was 14.2% (95% CI: 11.0%-18.2%). Predictive value of positive test was 94.4% (95% CI: 85.5%-98.0%), and predictive value of negative test was 18.3% (95% CI: 17.3%-19.3%). The prevalence of PAD among multivessel disease (double and triple vessel) was higher (16.8%) than in those with single-vessel disease (14.2%), and this result is statistically significant (p=0.037).

Conclusion

The prevalence of PAD among the patients undergoing coronary artery angiograms in this study is 12.5%. The strong association between CAD and PAD is confirmed in our population, and prediction of CAD using PAD (ABPI<0.9) showed high specificity but low sensitivity. A severe form of CAD is likely in patients with PAD.

## Introduction

Atherosclerosis is a systemic pathophysiological process characterized by the formation of potentially harmful plaques within the arterial lumen. These plaques, known as atheroma, cause arterial stiffening and narrowing, thereby compromising blood circulation and oxygen delivery to major organ systems [1,2]. When the atherosclerotic process involves the peripheral arterial system, other arterial territories are often affected concurrently. Patients are typically asymptomatic in the early stages and may remain unaware of the condition. Early diagnosis is helpful, as it can prevent life-threatening complications such as myocardial infarction, stroke, and peripheral artery disease (PAD) [3].

PAD, more precisely referred to as lower extremity artery disease, results from the deposition of fibro-fatty plaque material within arterial lumens, leading to impaired perfusion of the lower limb muscles [4]. It is more prevalent in individuals over 60 years of age. Most patients remain asymptomatic; however, some develop exertional muscle pain that is relieved by rest, a condition known as intermittent claudication. In addition to muscle pain, patients may present with pale, shiny skin, loss of hair over the extremities, numbness, weakness, muscle wasting, brittle toenails, poor wound healing, and, in males, erectile dysfunction [5].

The major risk factors for PAD are like those for coronary artery disease (CAD) and stroke, although their relative contributions may differ. Smoking is a particularly strong risk factor for PAD, along with diabetes mellitus and other emerging risk factors [6]. PAD is closely associated with concomitant coronary and cerebrovascular diseases. The ankle-brachial pressure index (ABPI) is a non-invasive method used for the diagnosis of PAD and monitoring treatment response. It is defined as the ratio of the highest systolic blood pressure measured at the ankle to the highest systolic blood pressure measured in the brachial artery, obtained in the supine position using a handheld Doppler ultrasound probe, which is considered the gold standard [7]. Additional diagnostic modalities include duplex ultrasonography, contrast angiography, computed tomography angiography, and magnetic resonance angiography.

ABPI demonstrates approximately 90% sensitivity and 98% specificity for the detection of PAD in patients with more than 50% arterial stenosis on angiogram [8]. Numerous studies have established ABPI as a strong indicator of diffuse atherosclerosis and an independent predictor of cardiovascular risk, including increased total and cardiovascular mortality. Recent guidelines from the European Society for Vascular Surgery indicate that an ABPI≤0.9 is associated with a two- to threefold increase in the risk of total mortality and cardiovascular death [9]. Conversely, an ABPI>1.4 is associated with increased arterial stiffness and higher cardiovascular mortality [10]. Currently, no local study has investigated the association between PAD and CAD.

Given that PAD is a marker of generalized atherosclerosis, accurate and timely detection using ABPI is essential, particularly in patients with atherosclerotic cardiovascular risk factors undergoing a coronary angiogram. Early identification and management may help prevent the progression of PAD during its asymptomatic stages [11,12]. However, due to the availability of Doppler-based ABPI measurement and awareness, PAD remains underdiagnosed. In a study conducted in Sri Lanka, only 4.1% of participants were aware of PAD, and this awareness was lower than that of other cardiovascular diseases such as cerebrovascular accidents (67.3%) and myocardial infarction (57.6%) (p<0.001) [13]. Similarly, in the Gampaha district of Sri Lanka, PAD was found to be a hidden disease with an age-sex standardized prevalence of 3.6% [14]. No studies were conducted on PAD in Northern Sri Lanka. Therefore, the aim of this study is to determine the prevalence of PAD, which is detected by ABPI among patients undergoing a coronary angiogram, describe the risk factors for PAD, and find out the association between CAD and PAD.

## Materials and methods

It was a cross-sectional analytical study conducted in a hospital setting, with hypotheses to be tested by collecting data from patients admitted to the cardiology ward and the coronary care unit of the Teaching Hospital, Jaffna, for a coronary angiogram. The study period was from October 2022 to May 2023. The Ethical Review Committee, Post Graduate Institute of Medicine, Faculty of Medicine, University of Colombo, approved the study protocol (ERC/PGIM/2022/006).

The following formula was used to calculate the sample size [15]: \[N = \frac{z^{2} p(1-p)}{d^{2}}\]​, where N = needed size of the sample, z = 95% confidence level (standard value of 1.96), p = prevalence of PAD among our population, 50% considered as estimated P, and d = margin of precision (5%). Therefore, the sample size was 427 after inflating to compensate for a 10% non-response rate.

We recruited 431 patients during the study period (consecutive sampling). The interviewer administered a questionnaire (pretested) translated into all three languages (Sinhala, Tamil, and English) for data collection from the patients, and a data extraction sheet was prepared to extract any relevant data from records according to specific objectives. The chief investigator and two pre-intern demonstrators, attached to the professorial medical unit, were trained by the chief investigator and helped to collect the data.

The patients were given enough time to read the participant’s information sheet and questions. The participant information sheet was presented orally to the illiterate participants. Patients who refused to give consent were not included in the study, and they were managed as usual patients with the same quality of care. All patients were allowed to withdraw their consent at any point of time during the study period, and their data were eliminated from the study.

Diagnosis of PAD and ABPI measurement

Diagnosis of PAD was based largely on clinical presentation and ABPI measurement. From the clinical history of cramping hip; thigh or calf pain; numbness or weakness of the lower limbs during exertion, which is relieved with the rest; poorly healing toe or foot wounds; erectile dysfunction in men, particularly with diabetes mellitus, and on physical examination, shiny skin due to loss of hair over extremities, poor toenail growth, absent peripheral pulses (dorsalis pedis, posterior tibial), and gangrenous tissue over the lower limbs are the pointers toward the clinical diagnosis of the lower limb arterial disease.

Exclusion criteria

During the data collection, patients who presented with severe upper or lower limb edema, had undergone amputation of the upper or lower limbs, were on limb prostheses, and patients with end-stage chronic kidney disease who were on hemodialysis were excluded from the study.

The ABPI was measured by the principal investigator using a standardized protocol. After obtaining consent, participants were asked to lie supine on an examination bed for at least 10 minutes before the measurements were taken. Brachial systolic blood pressure was measured in both arms using an appropriately sized blood pressure cuff. The brachial artery pulse was identified, and a small amount of ultrasound gel was applied over the artery. A handheld Doppler probe was positioned at an angle of 45°-60° to obtain an audible arterial signal. The cuff was inflated until the Doppler signal disappeared and then slowly deflated until the signal reappeared. The corresponding systolic pressure was recorded. The procedure was repeated for the contralateral arm, and the higher of the two brachial systolic pressures was used for the ABPI calculation. Ankle systolic pressures were measured in both legs using a blood pressure cuff placed just above the ankle. Ultrasound gel was applied over the posterior tibial artery and dorsalis pedis artery. Using the Doppler probe, the systolic pressure of each artery was measured by inflating the cuff until the arterial signal disappeared and then slowly deflating it until the signal returned. For each leg, the higher systolic pressure obtained from either the posterior tibial or dorsalis pedis artery was recorded as the ankle systolic pressure.

The ABPI was calculated separately for each leg using the following formula:

\[ \mathrm{ABPI} = \frac{\text{Highest ankle systolic pressure (for the respective leg)}}{\text{Highest brachial systolic pressure}} \]

The ABPI was calculated for each leg, and the lower value was considered for analysis. An abnormal value in either leg indicates PAD.

PAD diagnostic criteria

Measurement of ABPI was performed to confirm the diagnosis. In this study, the ABPI value was measured with the conventional Doppler method. ABPI between 1.00 and 1.40 is considered normal. A value of 0.9 or less is taken as abnormal. ABPI less than 0.5 is considered as severe PAD [16].

CAD diagnostic criteria

CAD was diagnosed based on visual estimation of ≥70% stenosis in at least one major epicardial coronary artery, performed by an experienced interventional cardiologist attached to the unit. The term "significant narrowing" refers to a diameter stenosis of over 70% in any major epicardial vessel in the "worst" angiographic view. Depending on the number of vessels affected, patients are categorized as having single-vessel, double-vessel, or triple-vessel disease [17].

Other variables

Moreover, BMI was also included using the Diabetes Association of Sri Lanka criteria, and data on diabetes mellitus, hypertension (HT), and metabolic syndrome were extracted from participants’ medical records. The diagnosis of diabetes mellitus and metabolic syndrome was based on the criteria established by the Diabetes Association of Sri Lanka. HT was defined as a documented diagnosis of HT in the medical records, current use of antihypertensive medications, or a recorded blood pressure meeting the accepted diagnostic criteria for HT (>140/90 mmHg) [18].

Statistical analysis

All collected data were entered into the Statistical Package for the Social Sciences (SPSS) software, version 30.0 (IBM Corp., Armonk, NY, USA), and used for analysis after cleaning and coding. The prevalence of PAD was presented as percentages with 95% CI. Descriptive statistics were used to present the distribution of variables. Continuous data were presented using mean ± SD, while categorical data were presented using percentages.

Associations between variables were assessed using a chi-square test, considering a p-value less than 0.05 as statistically significant. The strength of association between each variable and the expected outcome was calculated using unadjusted ORs with 95% CIs. WINPEPI updated: computer programs were used to calculate sensitivity, specificity, accuracy, positive predictive value, and negative predictive value for PAD in predicting CAD [19].

## Results

Out of 431 patients who underwent a coronary angiogram, most of them were male (74.0%) and studied only up to primary education (64.7%). Another 82 (19.0%) patients studied up to the ordinary level, and 39 (9.0%) completed tertiary education. The mean age of participants was 59.34 ± 10.19 years, with a minimum of 31 and a maximum of 81 years (Table 1).

**Table 1:** Sociodemographic characteristics of participants who underwent a coronary angiogram (n=431)

Characteristics			
		Mean ± SD	Minimum-maximum
Age		59.34 ± 10.19	31-81
	Categories	Number	Percentage
Sex	Male	319	74
	Female	112	26
Education	Primary education	279	64.7
	Up to O/L	82	19
	Up to A/L	31	7.2
	Tertiary	39	9

Among 431 participants, 30 (7.0%) were current smokers and 118 (27.4%) were ex-smokers. In further analysis, 73 (17.0%) people smoked less than 10 pack-years, another 60 (13.9%) smoked 11-30 pack-years, and 15 (3.5%) smoked more than 30 pack-years. Out of 431 patients, 98 (22.7%) consumed alcohol in moderate amount, and another 21 (4.9%) of them consumed alcohol more than 14 units. Seven patents were vegetarian (1.6%). The majority (73.3%) of patients reported that they engaged in moderate-to-intense physical activity. Most patients were obese (42.9%, 95% CI: 38.3-47.6). Further 215 (49.9%) patients had metabolic syndrome and 200 (46.4%) had DM. One hundred and one (23.4%) patients had a family history of CAD, and 35 (8.1%) had diabetic nephropathy. The majority (55.2%) of patients were diagnosed with HT, and 60.3% had dyslipidemia. One hundred and twenty-one (28.1%) patients had high low-density lipoprotein level (Table 2).

**Table 2:** Lifestyle and clinical characteristics of the study participants (n=431) This table describes the factors commonly associated with peripheral artery disease. CAD, coronary artery disease; LDL, low-density lipoprotein.

Variable	Category	Number	Percentage
Smoking	Current smokers	30	7.0
	Ex-smokers	118	27.4
	Never smoked	283	65.7
Smoking in pack-years	Less than 10 pack-years	73	17.0
	11-30 pack-years	60	13.9
	Greater than 30 pack-years	15	3.5
Alcohol	Not taking	312	72.4
	Moderate (<14 units/week)	98	22.7
	Severe (>14 units/week)	21	4.9
Diet	Vegetarian	7	1.6
	Non-vegetarian	424	98.4
Physical activity	Mild intense	31	7.2
	Moderate intense	316	73.3
	Vigorous	84	19.5
BMI in kg/m^2^	Underweight (<18.5)	35	8.1
	Normal weight (18.5-22.9)	125	29.0
	Overweight (23-24.9)	86	20.0
	Obese (≥25)	185	42.9
Metabolic syndrome	Present	215	49.9
Family history of CAD	Present	101	23.4
Diabetes mellitus	Present	200	46.4
Diabetic nephropathy	Present	35	8.1
Hypertension	Present	238	55.2
Dyslipidemia	Present	260	60.3
LDL	High (>130 mg/dL)	121	28.1

Prevalence of PAD among participants (ABPI less than 0.9)

Out of 431 participants who underwent a coronary angiogram, 54 (12.5%) had PAD (95% CI: 9.6-15.9), but no one had severe disease, as shown in Table 3. Around 3.2% of patients had moderately severe disease on the right limb, and 3.0% had moderately severe disease on the left.

**Table 3:** Comparison severity of PAD based on ABPI on both limbs ABPI, ankle-brachial pressure index; PAD, peripheral artery disease.

Severity	Right		Left	
	No	Percentage	No	Percentage
Severe (ABPI<0.49)	0	0	0	0
Moderate (ABPI 0.5-0.79)	14	3.2	13	3.0
Some (ABPI 0.8-0.89)	18	4.2	19	4.4
Acceptable (ABPI 0.9-1.0)	32	7.4	32	7.4
None (ABPI>1.0)	367	85.2	367	85.2

Out of 431 patients who underwent a coronary angiogram, 359 (83.3%) had CAD. Among 54 patients with PAD, 94.4% had CAD, while among 377 patients without PAD based on ABPI, 81.7% had CAD. This association was statistically significant (p=0.019). Patients who have PAD carry a 3.81 times higher risk (95% CI: 1.28-15.28) of having CAD as well.

Diagnostic performance analysis

Specificity of PAD for predicting CAD was 95.8% (95% CI: 88.4%-98.6%), sensitivity was 14.2% (95% CI: 11.0%-18.2%), and the area under the ROC curve was 55.0% (95% CI: 52.1%-58.0%). Predictive value of positive test was 94.4% (95% CI (Fleiss-Levin-Paik): 85.5%-98.0%), and predictive value of negative test was 18.3% (95% CI (Fleiss-Levin-Paik): 17.3%-19.3%). Percentage agreement (index of validity) was 27.8% (without allowing for chance agreement) (Table 4).

**Table 4:** Association of coronary artery disease with PAD and prediction of CAD based on ABPI<0.9 Chi square=5.516, df=1, p=0.019, OR=3.81 (1.28-15.78) ABPI, ankle-brachial pressure index; CAD, coronary artery disease; PAD, peripheral artery disease.

		CAD		Total	Sensitivity	Specificity	Positive predictive value	Negative predictive value
		Yes	No		14.21% (95% CI: 11.0%-18.2%)	95.83% (95% CI: 88.4%-98.6%)	94.4% (95% CI: 85.5%-98.0%)	18.3% (95% CI: 17.3%-19.3%)
PAD	Yes	51 (94.4%)	3 (5.6%)	54 (100%)				
	No	308 (81.7%)	69 (18.3%)	377 (100%)				
Total		359 (83.3%)	72 (16.7%)	431 (100%)				

Among 110 patients with triple-vessel disease, 19.1% had PAD. Similarly, among 115 patients with double-vessel disease, 9.5% had PAD. The prevalence of PAD among multivessel disease was higher (16.8%) than in those with single-vessel disease (14.2%) and in those with minor CAD (6.8%, p=0.037) (Table 5).

**Table 5:** Severity of CAD with PAD (n=431) Pearson chi-square=10.226, df=4, p=0.037 CAD, coronary artery disease; PAD, peripheral artery disease.

Coronary angiogram results	PAD		OR with 95% CI
	Yes	No	
Single-vessel disease	19 (14.2%)	115 (85.8%)	6.11 (1.05-131.58)
Double-vessel disease	10 (9.5%)	95 (90.5%)	3.89 (0.62-87.43)
Triple-vessel disease	21 (19.1%)	89 (80.9%)	8.73 (1.29-370.63)
Minor CAD	3 (6.8%)	41 (93.2%)	2.71 (0.27-73.07)
Normal coronary angiogram	1 (2.6%)	37 (97.4%)	
Total	54 (12.53%)	377 (87.47%)	431

## Discussion

This cross-sectional analytical study aimed to determine the prevalence of PAD, as detected by ABPI, among patients undergoing a coronary angiogram. The study also evaluated factors associated with PAD and examined the association between CAD and PAD. We recruited a total of 431 patients who were admitted for coronary angiograms at the Teaching Hospital, Jaffna, during the study period.

The prevalence of PAD was 12.5% (95% CI: 9.6-15.9), higher than the prevalence reported in the general population of the Gampaha district of Sri Lanka, which was 3.6% (95% CI: 2.9%-4.3%) [14]. This finding is not unexpected, as patients undergoing a coronary angiogram generally have a higher risk of PAD. Interestingly, a similar prevalence rate (12.8%) of unrecognized PAD was reported in Jordan Hospital [20].

Further, no significant difference between men and women (13.4% vs. 11.7%, p=0.485) was reported in a similar study [20]. This study also failed to show any difference between sex (p=0.732). Some studies have shown a unilateral course of disease [6]. However, no significant difference was observed in ABPI between both lower limbs in this study, as illustrated in Table 3.

The Comprehensive Heart and Multidisciplinary Limb Preservation Outreach Networks (CHAMPIONS) programs screened for risk factors for PAD and found a distinct PAD risk profile among Indians, such as diabetes (p=0.002), hyperlipidemia (p=0.006), HT (p=0.047), and a family history of vascular disease (p<0.001). They were carrying a higher risk for PAD (p<0.001) than non-Indian Asian participants [21]. In this study, most participants had dyslipidemia (60%), HT (55.2%), metabolic syndrome (49.9%), and high BMI (62.9%). Further, a study in the Middle East revealed that smoking (OR: 1.3, 95% CI: 0.7-2.1) is an independent risk factor for PAD [20], and in this study, 7% of patients were current smokers and 27.4% were ex-smokers. Furthermore, the current study also demonstrated the fact that with increasing intensity of physical activity among participants, the proportion of PAD decreased from 29.0% to 2.4% (p<0.001). This result is in par with findings from longitudinal studies, suggesting that more intense physical activity is associated with a lower risk of developing PAD [22]. In this study, out of 238 patients with HT, 14.7% had PAD, whereas among 193 patients without HT, only 9.8% had PAD. Several epidemiological studies have consistently shown that patients with HT had a higher prevalence of PAD [23].

The strong relationship between PAD, as detected by ABPI, and CAD, confirmed by coronary angiogram, was observed in this study, depicted in Table 4 (p=0.019), and a similar pattern was noted in a study conducted in a slightly different setting/population by Kim et al. [9] and Saleh et al. [20]. Patients with PAD exhibit a 3.8 times risk (95% CI: 1.3-15.8) of having CAD. Further analysis also revealed that the specificity of PAD in predicting CAD was 95.8% (95% CI: 88.5%-98.6%), with a positive predictive value of 94.4% (95% CI: 85.5%-98.0%), which is greater than that of a similarly aimed study, which reported specificity of 82.1% [24]. However, sensitivity was 80.0% (95% CI: 70.8%-90.4%) in that particular study, whereas this study showed a sensitivity of 14.2% (95% CI: 11.0%-18.2%). This is because the prevalence of PAD was 12.5% compared with 62.8% in the previous study [24]. Similarly, a study conducted in Brazil indicated that ABPI could predict significant CAD in patients with ABPI≤0.87, with a positive predictive value of 75.9% and a negative predictive value of 71.6% [25]. However, it is questionable to use ABPI in patients with high risk factors to detect a major part of asymptomatic CAD at an early stage due to its lower sensitivity. However, it is reasonable to do ABPI in patients undergoing a coronary angiogram to detect asymptomatic PAD, as many studies have suggested that asymptomatic PAD is more prevalent than symptomatic PAD [6-8] among patients undergoing a coronary angiogram. Due to the high specificity observed, the chances of false positives for CAD are less (the predictive value of a positive test was 94.4%).

Further, our analysis also showed that the patients with PAD had a more severe form of CAD such as multivessel coronary disease, as shown in Table 5. This finding is par in line with a previous study, which revealed that multivessel CAD had a two times higher risk of being diagnosed with previously unrecognized PAD than those with single-vessel CAD (OR: 2.0, 95% CI: 1.0-4.0) [26].

In summary, the prevalence of asymptomatic PAD in our population is comparable to the prevalence reported in Arab populations, the cohorts in Germany, Switzerland, France, Japan, and Turkey [27-30], and it is reasonable to do ABPI in patients undergoing a coronary angiogram to detect asymptomatic PAD.

Limitations

This study has several limitations. First, its design and sample size may limit the generalizability of the findings to the broader population. Second, the cross-sectional nature of the study precludes establishing a causal relationship between the ABPI and CAD. Third, potential measurement variability in the Doppler-based ABPI assessment may have influenced the accuracy of PAD detection, even though inter-observation variability was reduced to some extent, as all ABPI measurements were performed by the principal investigator. Additionally, residual confounding from unmeasured risk factors such as vessel stiffness and medial artery calcification, both of which alter distensibility, cannot be excluded. So, we recommend a prospective study with control in the future to minimize the limitations observed in this study.

## Conclusions

Unrecognized PAD was common among patients undergoing a coronary angiogram. A significant association was observed between PAD, assessed by the ABPI, and the presence of CAD. Contributing factors for PAD such as smoking, alcohol consumption, physical inactivity, obesity, metabolic syndrome, diabetes mellitus, HT, and dyslipidemia were prevalent in our population.

Although PAD demonstrated high specificity for predicting CAD with positive predictive value (94.4%), its negative predictive value was low (18.3%); as such, general population screening for PAD to predict CAD is questionable in our population. Nevertheless, ABPI screening may serve as a simple, non-invasive, and useful tool for identifying previously unrecognized PAD among patients undergoing a coronary angiogram to detect asymptomatic PAD, as many studies have suggested that asymptomatic PAD is more prevalent than symptomatic PAD.
